# Uterine fluid proteomic and metabolomic profiles associated with soft-shelled egg formation in laying hens

**DOI:** 10.3389/fvets.2026.1932663

**Published:** 2026-09-04

**Authors:** Kaiqi Weng, Maodou Xu, Lina Song, Qi Xu, Yu Zhang

**Affiliations:** 1 Key Laboratory for Evaluation and Utilization of Poultry Genetic Resources of Ministry of Agriculture and Rural Affairs, Yangzhou University, Yangzhou, China; 2 Institute of Animal Science and Veterinary Medicine, and Zhuanghang Integrated Experiment Station, Shanghai Academy of Agricultural Sciences, Shanghai, China

**Keywords:** egg quality, laying hens, metabolomics, proteomics, soft-shelled egg, uterine fluid

## Abstract

**Introduction:**

Soft-shelled eggs are a major eggshell defect in poultry production, yet the underlying mechanisms of their formation remain unclear. The objective of this study was to characterize biological changes associated with soft-shelled egg formation in laying hens by integrating quantitative proteomics and untargeted metabolomics analyses of uterine fluid (UF), combined with morphological and physiological assessments.

**Methods:**

Normal-shelled egg-producing hens (NE) and soft-shelled or shell-less egg-producing hens (SE) were selected from Langshan laying hens maintained under the same feeding and management conditions. Egg quality traits, serum biochemical indicators, duodenal and uterine morphology, TMT-based proteomics, and LC–MS-based untargeted metabolomics of UF were analyzed.

**Results:**

Compared with the NE group, the SE group exhibited significantly reduced eggshell thickness and disrupted eggshell ultrastructure (*p* < 0.05). No significant differences were detected in the measured serum calcium-related indicators or duodenal morphology between the two groups (*p* > 0.05). In contrast, the NE group showed significantly greater endometrial villus length, uterine mucosal fold height, and mucosal fold area than the SE group (*p* < 0.05). Proteomic and metabolomic analyses revealed changes in UF protein and metabolite profiles, and the differential proteins and metabolites were mainly enriched in pathways related to amino acid metabolism and mitochondrial energy metabolism, including aminoacyl-tRNA biosynthesis and L-cysteine-related metabolism.

**Conclusion:**

Soft-shelled egg formation in laying hens was associated with compromised uterine morphology and changes in the UF metabolic microenvironment. These findings provide insight into uterine fluid protein and metabolite profiles related to eggshell defects and identify candidate pathways for future validation.

## 1 Introduction

Eggshell quality is a critical determinant of egg safety, hatchability, and economic return in the poultry industry (1). Inferior eggshells, including soft-shelled eggs, are characterized by impaired shell structure and reduced mechanical strength, increasing the risk of breakage and causing economic losses in poultry production (2, 3). Soft-shelled eggs are particularly problematic because their poor shell integrity increases the risk of damage and reduces their suitability for incubation (2). Therefore, improving eggshell quality is essential for enhancing production efficiency and economic sustainability in poultry systems.

Egg formation is a highly coordinated biological process. Following ovulation, the yolk sequentially passes through the infundibulum, magnum, isthmus, and uterus of the oviduct, forming a complete egg within approximately 24–25 h, of which about 20 h are devoted to eggshell formation in the uterus (4, 5). The eggshell is composed mainly of approximately 95% calcium carbonate and 3.5% organic matrix proteins (6); however, the constituent ions (Ca^2+^ and HCO₃^−^) are not stored in the uterus and must be continuously supplied from the bloodstream. Once the egg enters the uterus, uterine tissue is stimulated to initiate active calcium secretion (7). Calcium ions are transported from the blood into the mucosal epithelium of the eggshell glands primarily via transient receptor potential channels (8). Intracellular storage, transport, and regulation of calcium concentration are mediated by calcium-binding proteins and the endoplasmic reticulum (9, 10), after which calcium is secreted into the uterine fluid through plasma membrane calcium ATPases and sodium-calcium exchangers. These processes provide the essential substrates for the efficient and orderly deposition of the eggshell (11, 12). Transcriptomic studies have also identified uterine genes and biological pathways involved in calcium transport and eggshell biomineralization (13). Previous studies have demonstrated that interactions between proteins and ions in the uterine fluid play a crucial role in determining eggshell ultrastructure and mechanical strength (14). Therefore, investigating changes in the composition of uterine fluid is important for understanding molecular changes associated with eggshell formation.

A limited number of studies have investigated eggshell formation from the perspective of uterine fluid. Using quantitative proteomics, Marie et al. (15) reported that 64 proteins were associated with eggshell calcification in the uterine fluid of laying hens. However, previous studies on soft-shelled eggs have primarily focused on external factors such as stress, disease, and dietary nutrition, whereas local molecular changes associated with soft-shelled egg formation remain unclear (2). Therefore, the objective of the present study was to characterize differences in proteins and metabolites related to eggshell quality in uterine fluid using integrated quantitative proteomics and untargeted metabolomics. In addition, physiological and histological characteristics of hens producing soft-shelled eggs and normal eggs were systematically compared. The findings of this study provide a molecular basis for understanding eggshell defects and identify candidate pathways for improving eggshell quality in poultry production.

## 2 Materials and methods

### 2.1 Animals and sample collection

All experimental procedures involving animals were conducted in accordance with the Regulations on the Administration of Laboratory Animals issued by the State Council of the People’s Republic of China (2017). The study protocol was reviewed and approved by the Institutional Animal Care and Use Committee of Yangzhou University (Permit Number: YZUDWSY20250285, Jiangsu, China). Langshan laying hens (*Gallus gallus*) were raised at a Langshan Chicken Breeding Farm in Rudong County, Jiangsu, China. The Langshan chicken is a traditional indigenous Chinese breed originating from Rudong County and is classified as a local genetic resource under national conservation programs. In this experiment, Langshan laying hens were individually caged and maintained under a 16 h light:8 h dark photoperiod (16L:8D). All hens were provided ad libitum access to the same basal layer diet and water throughout the experiment, and no dietary supplementation or nutritional intervention was applied. A two-week laying observation period was initiated when the hens were 25 weeks old, at which time the flock laying rate was 67.4%, corresponding to the early laying stage. Exact individual days in lay prior to the observation period were not available from the farm records; therefore, laying stage was defined according to flock age and laying rate. Hens in the NE and SE groups were selected from the same age-matched flock and were maintained under identical feeding and management conditions. During this observation period, six hens consistently producing normal-shelled eggs with regular shape and normal eggshell cuticle color were selected as the normal egg group (NE), whereas six hens recurrently producing abnormally shelled eggs during the two-week observation period were assigned to the soft-shelled egg group (SE). These hens could produce either soft-shelled or, occasionally, shell-less eggs at different laying events, with soft-shelled eggs representing the predominant abnormal phenotype. Normal-shelled eggs were identified by routine visual inspection as having an intact hard shell, a regular oval shape without obvious deformation, and no obvious abnormality in eggshell color. Soft-shelled and shell-less eggs were readily distinguished visually by the presence of a soft, incompletely mineralized shell or the absence of a calcified shell, respectively. Before euthanasia, venous blood was collected from all 12 hens, and serum samples were separated by centrifugation and stored at −80 °C. Following euthanasia by cervical dislocation, uterine tissues, uterine fluid, and duodenum samples were collected. Sampling was performed after the routine morning egg collection, beginning at approximately 09:00. No egg was present in the uterus at the time of sampling. More than 2 mL of uterine fluid was directly aspirated from the uterine lumen of each hen without dilution and subsequently used for proteomic and metabolomic analyses. The samples were rapidly frozen in liquid nitrogen and stored at −80 °C until further analysis.

Routine production records from the Langshan chicken breeding farm were also used to evaluate the occurrence of soft-shelled eggs during the early, middle, and late laying stages. Soft-shelled egg production rate was calculated as the number of soft-shelled eggs divided by the total number of eggs produced during the corresponding laying stage × 100%.

### 2.2 Egg trait measurements and eggshell ultrastructure analysis

Twenty eggs per group were collected for egg quality measurements, and all measurements were performed on the day of sampling. In the SE group, all 20 eggs used for these analyses were soft-shelled eggs; shell-less eggs were excluded from eggshell thickness and ultrastructural measurements because they lacked a calcified shell. Egg weight, yolk color, yolk weight, Haugh unit, and albumen height were determined using a multifunctional egg quality analyzer (EMT-5200; Robotmation Co., Ltd., Tokyo, Japan). Egg shape index (length/width) was calculated using a caliper. Eggshell breaking strength was measured using an Eggshell Force Gauge (EFG-0503; Robotmation Co., Ltd., Tokyo, Japan), and eggshell thickness was determined with a dial pipe gauge (EFG-1061A; Robotmation Co., Ltd., Tokyo, Japan). Eggshell color was evaluated using the L*a*b* color system. Eggshell ultrastructure was examined using a scanning electron microscope (JSM-6390LV, JEOL Ltd., Tokyo, Japan). Mammillary knob width, mammillary layer thickness, palisade layer thickness, and effective layer thickness were measured from cross-sectional scanning electron micrographs. The palisade layer was measured separately from the upper boundary of the mammillary layer to the boundary of the vertical crystal layer. The effective layer was defined as the combined thickness of the palisade layer, vertical crystal layer, and cuticle.

### 2.3 Morphological observation and histological staining analysis

Uterine and duodenal tissues were embedded in paraffin, sectioned, and subjected to hematoxylin and eosin staining. Quantitative image analysis was conducted using Image-Pro Plus software (Media Cybernetics, Rockville, MD, USA). In the duodenum, villus height and crypt depth were measured across 10 randomly selected fields, and the villus height-to-crypt depth ratio was calculated. Uterine villus length was defined as the distance from the villus tip to the lamina propria. Mucosal fold height was determined as the vertical distance from the epithelial surface to the fold apex, and fold width was measured at the widest point perpendicular to the fold axis. Fold area was calculated by tracing the outline of each fold. Measurements were obtained from six biological replicates per group, with six observations recorded for each sample.

### 2.4 Enzyme-linked immunosorbent assay

Serum alkaline phosphatase (ALP), calcitonin (CT), parathyroid hormone (PTH), calcium (Ca), and phosphorus (P) concentrations were determined using ELISA kits (Jiancheng, Nanjing, China) according to the manufacturer’s instructions.

### 2.5 Proteomics analysis

Four biological replicates from each group (*n* = 4 per group) were used for TMT-based quantitative proteomic analysis. Proteins in uterine fluid were extracted using a lysis buffer containing 8 mol/L urea and 1% sodium dodecyl sulfate. Protein concentrations in the supernatant were determined using a bicinchoninic acid (BCA) protein assay kit (Thermo Fisher Scientific, Wilmington, DE, USA) according to the manufacturer’s instructions. Samples were diluted to a final concentration of 0.5 μg/μL with 1× TEAB, followed by reduction with 10 mM TCEP and alkylation with 25 mM CAA at 37 °C for 30 min. Pre-washed beads were then added and incubated at room temperature for 18 min. After incubation, the supernatant was discarded, the magnetic beads were washed several times, and 40 μL of 1× TEAB was added to resuspend the beads. Trypsin was added at a protein-to-trypsin ratio of 10:1, and samples were incubated at 37 °C for at least 4 h. Digestion was terminated by adding 5% TFA. TMT reagents were added to 100 μg of digested peptides and incubated at room temperature for 1 h. The reaction was quenched by adding ammonia solution. The labeled samples were pooled and dissolved in 100 μL of mobile phase A (100% water containing 0.1% formic acid). Following centrifugation at 14,000 × *g* for 20 min, the supernatant was collected and subjected to high-performance liquid chromatography fractionation. Mass spectrometry detection was performed using an ORBITRAP ECLIPSE mass spectrometer equipped with a FAIMS Pro Interface and Nanospray Flex ion source. RAW data files were processed and quantified using Proteome Discoverer software (v2.4.1.15; Thermo Fisher Scientific, MA, USA). The UniProt *G. gallus* database was employed for this analysis. Protein quantitative values were normalized using median normalization to minimize technical variation among samples. Differences in protein abundance between the NE and SE groups were assessed using Student’s *t*-test, and the resulting *p*-values were adjusted for multiple testing using the Benjamini–Hochberg false discovery rate (BH-FDR) procedure. Differentially abundant proteins (DAPs) were defined based on fold change thresholds (FC > 1.2 or < 0.83) and *p* < 0.05. Functional annotation was conducted using the Kyoto Encyclopedia of Genes and Genomes (KEGG; https://www.genome.jp/kegg/). Protein–protein interaction networks were analyzed using the STRING database, and network visualization was performed with the igraph package. Proteomics data are available via ProteomeXchange with identifier PXD078015.

### 2.6 Metabolomics analysis

All six biological replicates from each group (*n* = 6 per group) were used for untargeted metabolomic analysis. Metabolites were extracted by homogenizing samples in a methanol–chloroform–water solution with steel beads, followed by repeated grinding, ultrasonic treatment, and centrifugation (12,000 rpm, 4 °C, 10 min). The supernatant was collected, reconstituted in 50% acetonitrile containing an internal standard, and filtered for LC–MS analysis. LC analysis was performed using a Vanquish UHPLC system (Thermo Fisher Scientific, USA) equipped with an ACQUITY UPLC HSS T3 column (150 × 2.1 mm, 1.8 μm; Waters, USA) maintained at 40 °C. The flow rate and injection volume were set at 0.25 mL/min and 2 μL, respectively. Mobile phases consisted of 0.1% formic acid in water and acetonitrile for positive ion mode, and ammonium formate with acetonitrile for negative ion mode, with gradient elution applied. Mass spectrometry was conducted on a Q Exactive system (Thermo Fisher Scientific, USA) with an electrospray ionization source operating in both positive and negative modes. Data were acquired in Full MS-ddMS^2^ mode. Metabolites were annotated based on accurate mass, MS/MS spectra, and comparisons with public databases, including HMDB, MassBank, LipidMaps, mzCloud, and KEGG. Because authentic standards were not available for all metabolites, metabolite identities should be considered putative unless otherwise specified. Data were normalized using QC-based LOESS signal correction. Features with relative standard deviation (RSD) > 30% in QC samples were excluded. Differential metabolites were screened based on nominal *p* < 0.05 and VIP > 1 (OPLS-DA), with fold change (FC) used to describe the magnitude and direction of changes. *p*-values were additionally adjusted for multiple testing using the BH-FDR procedure. Pathway enrichment and topology analyses were performed using MetaboAnalyst, and metabolic pathways were annotated via KEGG.

### 2.7 Statistical analysis

Differences in egg traits and other non-omics variables between the NE and SE groups were analyzed using an independent-samples *t*-test in SPSS 25.0. Soft-shelled egg production rates among the early, middle, and late laying stages were analyzed using one-way ANOVA followed by Tukey’s multiple-comparison test. Data are presented as mean ± standard error of the mean (SEM), and *p* < 0.05 was considered statistically significant.

## 3 Results

### 3.1 Comparisons of egg quality traits

**Figure 1:**
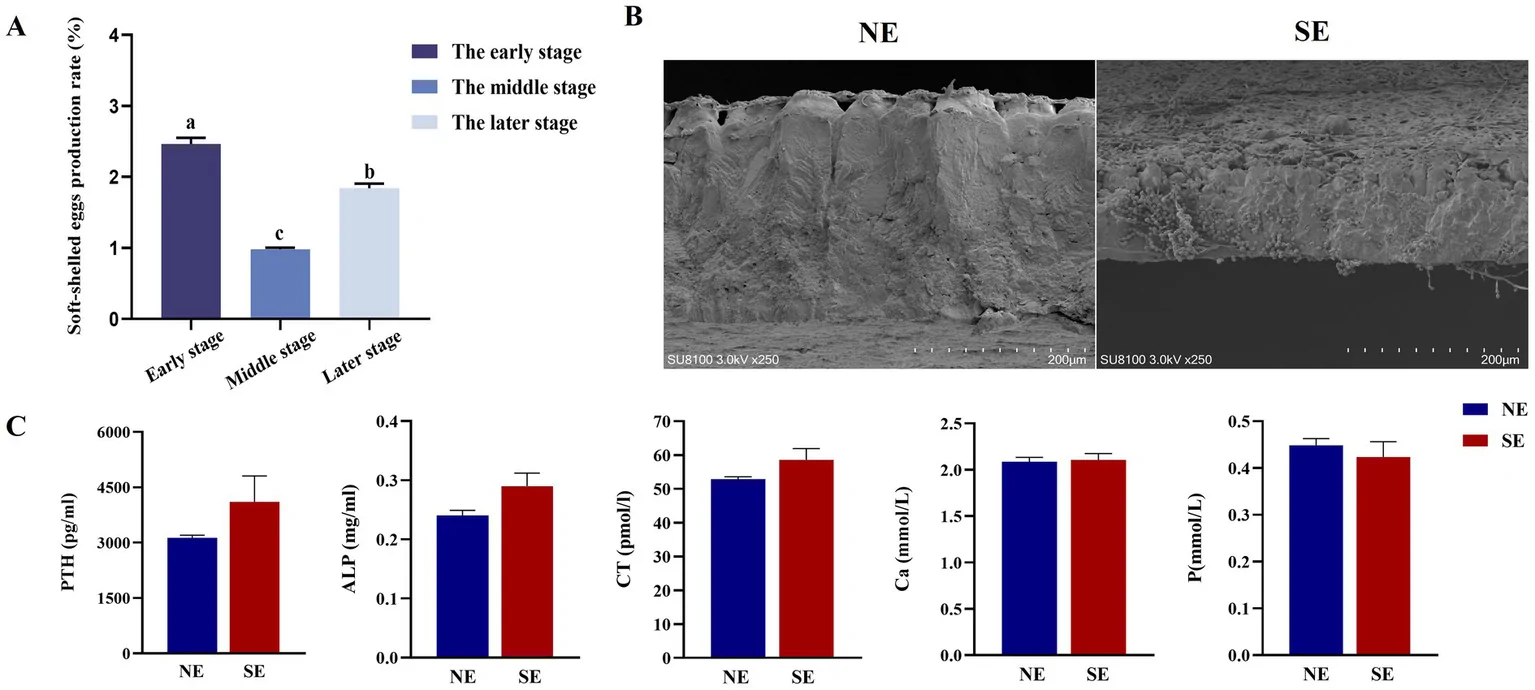
Eggshell quality characteristics and serum biochemical parameters in laying hens with normal and soft-shelled eggs. **(A)** Soft-shelled egg production rate during the early, middle, and late laying stages. Different lowercase letters indicate significant differences among laying stages (*p* < 0.05). **(B)** Representative scanning electron micrographs showing eggshell ultrastructure in the normal egg (NE) and soft-shelled egg (SE) groups. **(C)** Serum biochemical parameters in hens laying NE and SE.

The production rate of soft-shelled eggs was highest during the early laying stage (Figure 1A). Comparisons of egg quality traits in the NE and SE groups are provided in Table 1. Eggshell ultrastructure, including the mammillary, palisade, and effective layers, is shown in Figure 1B. No significant differences were detected between the NE and SE groups in egg weight, egg shape index, L* and b* values, yolk weight, yolk color, Haugh unit, or albumen height (*p* > 0.05). The a* value was significantly lower in the SE group than in the NE group (*p* < 0.05). Eggshell thickness, mammillary knob width, mammillary layer thickness, palisade layer thickness, and effective layer thickness were also significantly reduced in the SE group compared with the NE group (*p* < 0.05). Due to the extreme fragility of soft-shelled eggs, eggshell breaking strength could not be measured in the SE group. Collectively, these results indicate that soft-shelled eggs exhibited markedly reduced eggshell thickness and impaired shell ultrastructure, whereas most internal egg quality traits did not differ significantly between the two groups.

**Table 1:** Comparisons of egg quality traits in NE and SE groups.

Index		NE group	SE group
Egg weight (g)		41.75 ± 3.45	38.58 ± 5.27
Egg shape index		1.37 ± 0.05	1.39 ± 0.02
Eggshell breaking strength (kg/cm^2^)		4.53 ± 0.62	NA
Eggshell thickness (mm)		0.39 ± 0.03	0.29 ± 0.01*
Eggshell color	L*	62.15 ± 1.32	64.59 ± 5.79
	a*	11.63 ± 1.32	6.86 ± 1.39*
	b*	11.08 ± 2.21	8.68 ± 3.64
Yolk weight (g)		11.85 ± 0.51	11.3 ± 1.28
Yolk color		7.28 ± 0.39	6.56 ± 1.21
Haugh unit		79.68 ± 3.07	83.80 ± 5.20
Albumen height (mm)		5.43 ± 0.51	5.90 ± 0.88
Mammillary knob width (μm)		42.63 ± 8.98	25.84 ± 3.13*
Mammillary layer thickness (μm)		42.48 ± 3.85	20.52 ± 4.73*
Palisade layer thickness (μm)		213.47 ± 2.35	87.93 ± 3.20*
Effective layer thickness (μm)		239.58 ± 1.93	92.41 ± 6.77*

Results are reported as the mean ± SEM. *Means within a row significantly differ compared to the NE group (*p* < 0.05). All 20 eggs used for egg quality and eggshell ultrastructural measurements in the SE group were soft-shelled eggs; no shell-less eggs were included. Due to the extreme fragility of soft-shelled eggs, eggshell breaking strength could not be measured in the SE group.

### 3.2 Comparisons of serum biochemical indicators and duodenal morphology

**Figure 2:**
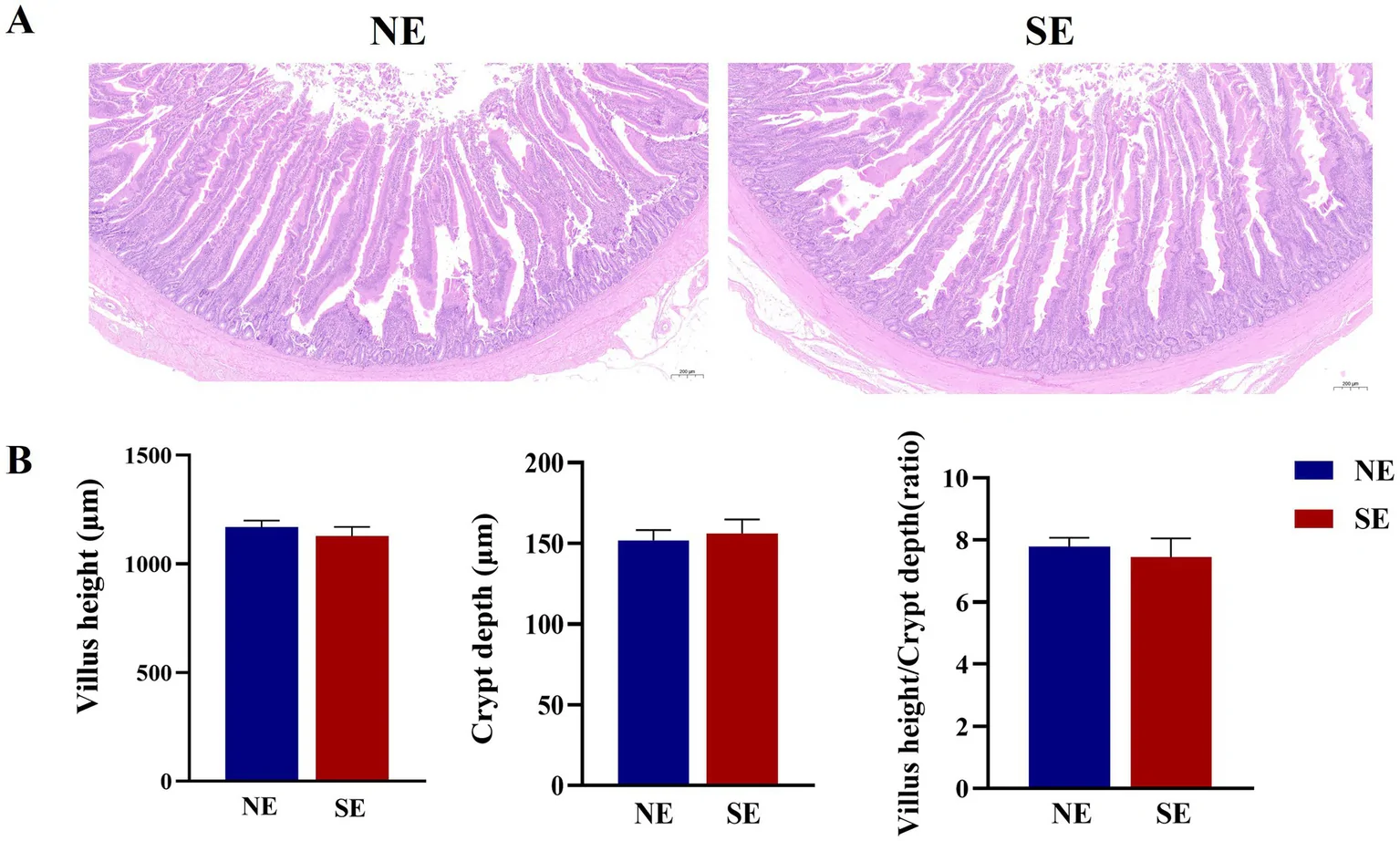
Duodenal morphology in laying hens with normal and soft-shelled eggs. **(A)** Representative histological sections of the duodenum from hens laying NE and SE. **(B)** Quantitative analysis of duodenal villus height, crypt depth, and villus height-to-crypt depth ratio in the NE and SE groups.

As shown in Figure 1C, serum biochemical indicators, including parathyroid hormone, alkaline phosphatase, and calcitonin, did not differ significantly between groups (*p* > 0.05). Similarly, no significant differences were observed in serum calcium or phosphorus concentrations (*p* > 0.05). Histological examination of the duodenum (Figure 2) revealed no significant differences in villus height, crypt depth, or the villus height-to-crypt depth ratio between the NE and SE groups (*p* > 0.05). Taken together, these results indicate that no significant differences were detected in the measured systemic calcium-related indicators or duodenal morphology between the NE and SE groups.

### 3.3 Comparisons of histological characteristics of chicken uterus

**Figure 3:**
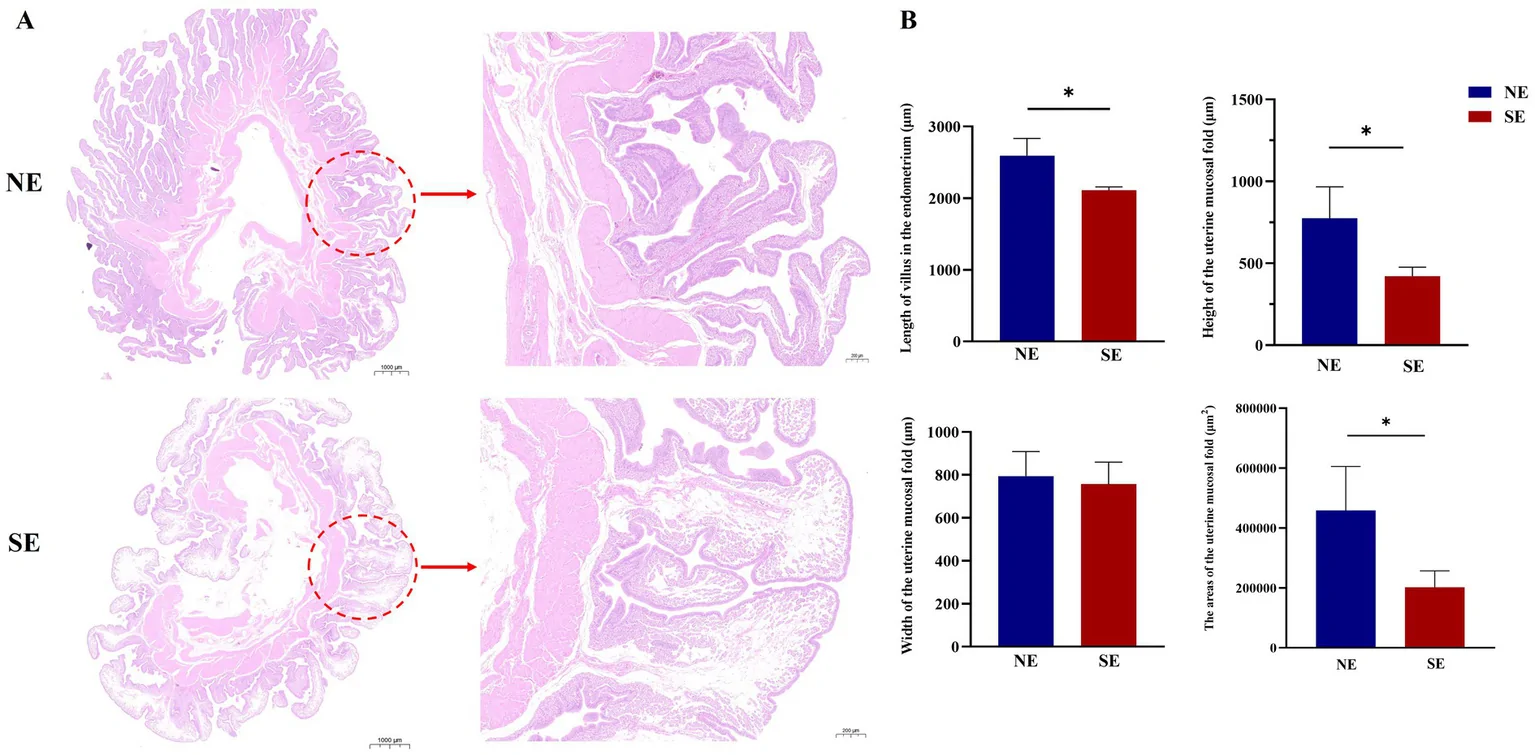
Histological characteristics of the uterus in laying hens with normal and soft-shelled eggs. **(A)** Representative histological sections of uterine tissues from hens laying NE and SE. **(B)** Quantitative analysis of uterine morphological parameters, including endometrial villus length, mucosal fold height, mucosal fold width, and mucosal fold area.

Compared with the SE group (Figure 3), the NE group exhibited significantly greater endometrial villus length, uterine mucosal fold height, and mucosal fold area (*p* < 0.05). In contrast, no significant difference was observed in mucosal fold width between the two groups (*p* > 0.05). Collectively, these results indicate marked alterations in uterine villus and mucosal fold morphology in hens producing soft-shelled eggs.

### 3.4 Untargeted metabolomic profiling of uterine fluid

**Figure 4:**
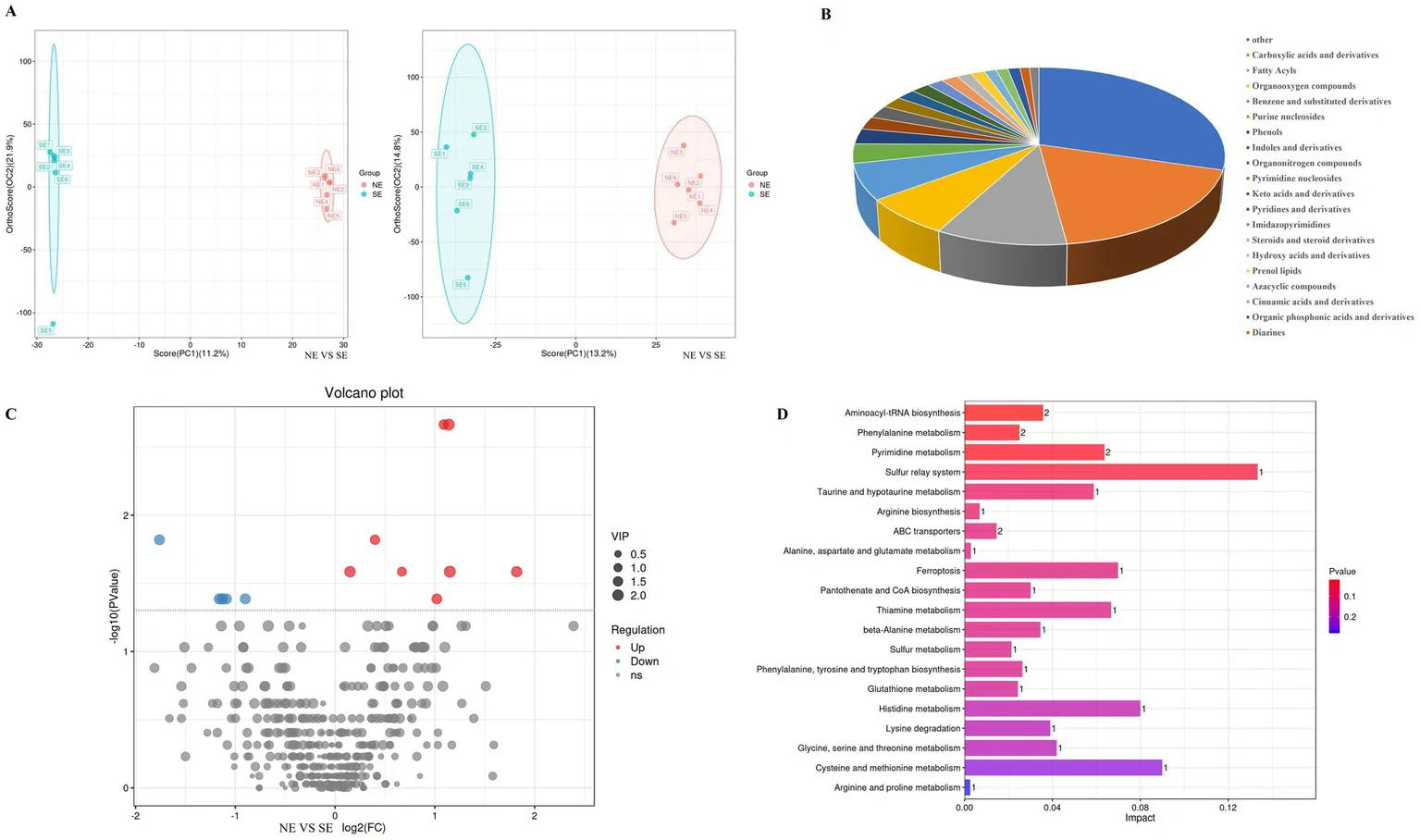
Untargeted metabolomic profiling of uterine fluid from laying hens with normal and soft-shelled eggs. **(A)** OPLS-DA score plots of uterine fluid metabolites from the NE and SE groups in positive and negative ion modes. **(B)** Classification of identified uterine fluid metabolites into major chemical categories. **(C)** Volcano plot showing differentially expressed metabolites between the NE and SE groups. Fold change was calculated as NE/SE; thus, red ‘Up’ points indicate metabolites with higher abundance in NE than SE, whereas blue ‘Down’ points indicate metabolites with lower abundance in NE than SE. **(D)** KEGG pathway enrichment analysis of differentially expressed metabolites.

OPLS-DA analysis revealed a clear separation between SE and NE groups in both positive and negative ion modes, indicating distinct untargeted metabolomic profiles of uterine fluid between the two groups (Figure 4A). A total of 429 metabolites were identified, the majority of which were shared between groups. These metabolites were classified into 25 categories, with carboxylic acids and derivatives (78), fatty acyls (43), organooxygen compounds (16), benzene and substituted derivatives (17), purine nucleosides (15), and phenols (12) representing the predominant classes (Figure 4B). Overall, the uterine fluid metabolome was predominantly composed of carboxylic acids and derivatives, fatty acyls, organooxygen compounds, benzene derivatives, and purine nucleosides.

Differentially expressed metabolites (DEMs) were identified based on nominal *p* < 0.05 and variable importance in projection (VIP) > 1 (Supplementary Table S1), with fold change (FC) used to describe the magnitude and direction of changes. Compared with the NE group, 13 DEMs were identified in the SE group, including 5 upregulated and 8 downregulated metabolites (Figure 4C). The upregulated metabolites included L-cysteine, N-acetyl-L-glutamate 5-semialdehyde, ureidosuccinic acid, β-leucine, and apigenin, whereas 3-hydroxyphenylacetic acid, L-histidine, aminoadipic acid, 5-guanidino-3-methyl-2-oxopentanoate, prephenate, uridine, o-toluate, and salicylic acid were downregulated. KEGG pathway enrichment analysis revealed that these DEMs were primarily involved in amino acid-related metabolic pathways, including aminoacyl-tRNA biosynthesis, the sulfur relay system, cysteine and methionine metabolism, histidine metabolism, and thiamine metabolism (Figure 4D). Collectively, these results suggest that alterations in amino acid metabolism, including pathways involving L-cysteine, may be associated with soft-shelled egg formation.

### 3.5 Quantitative proteomic analysis of uterine fluid

**Figure 5:**
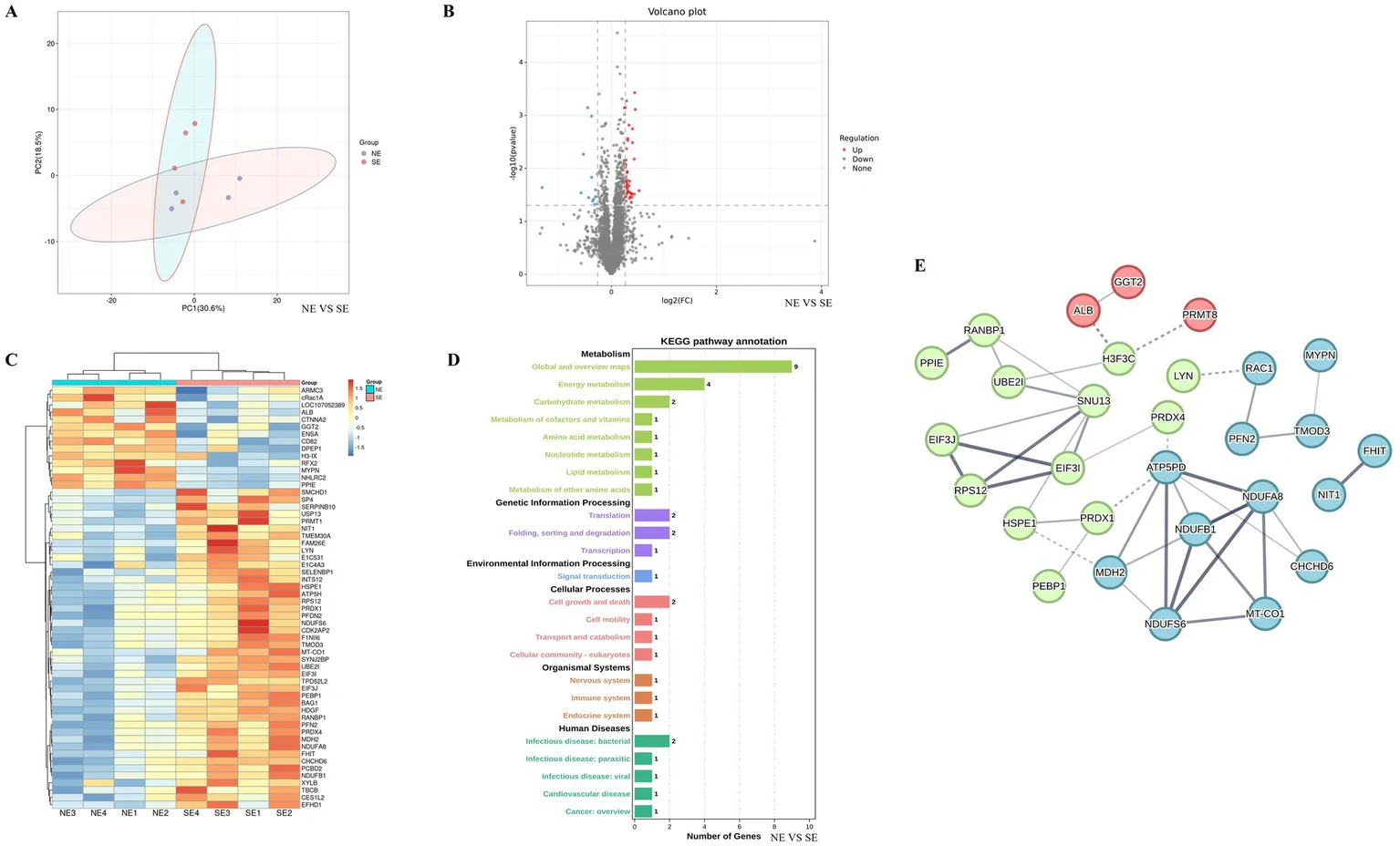
Quantitative proteomic profiling of uterine fluid from laying hens with normal and soft-shelled eggs. **(A)** Principal component analysis of uterine fluid proteomes from the NE and SE groups. **(B)** Volcano plot showing differentially abundant proteins (DAPs) between the NE and SE groups. **(C)** Hierarchical clustering heatmap of DAPs based on relative protein abundance. **(D)** KEGG pathway annotation of DAPs. **(E)** Protein–protein interaction network of DAPs constructed using the STRING database.

TMT-based quantitative proteomics was performed to characterize the protein profiles of uterine fluid from the NE and SE groups. Principal component analysis demonstrated a clear separation between the NE and SE proteomes (Figure 5A). Based on the criteria of fold change (FC) >1.2 or <0.83 and *p* < 0.05, a total of 58 differentially abundant proteins were identified between the two groups, including 44 upregulated and 14 downregulated proteins in the SE group (Figure 5B; Supplementary Table S2). Hierarchical clustering analysis further illustrated distinct protein abundance patterns between NE and SE samples (Figure 5C).

To explore the functional characteristics of the DAPs, KEGG pathway annotation was performed. KEGG pathway analysis indicated that DAPs were mainly associated with global and overview maps, energy metabolism, carbohydrate metabolism, and cell growth and death pathways (Figure 5D).

A protein–protein interaction (PPI) network of the DAPs was constructed using the STRING database and visualized with igraph software (Figure 5E). The major interaction clusters were enriched in the respiratory chain complex, proton-transporting ATP synthase complex, inner mitochondrial membrane protein complex, and mitochondrial outer membrane translocase complex, suggesting alterations in mitochondrial-related protein networks in the uterine fluid of SE hens.

### 3.6 Comprehensive analysis of proteomics and metabolomics

**Figure 6:**
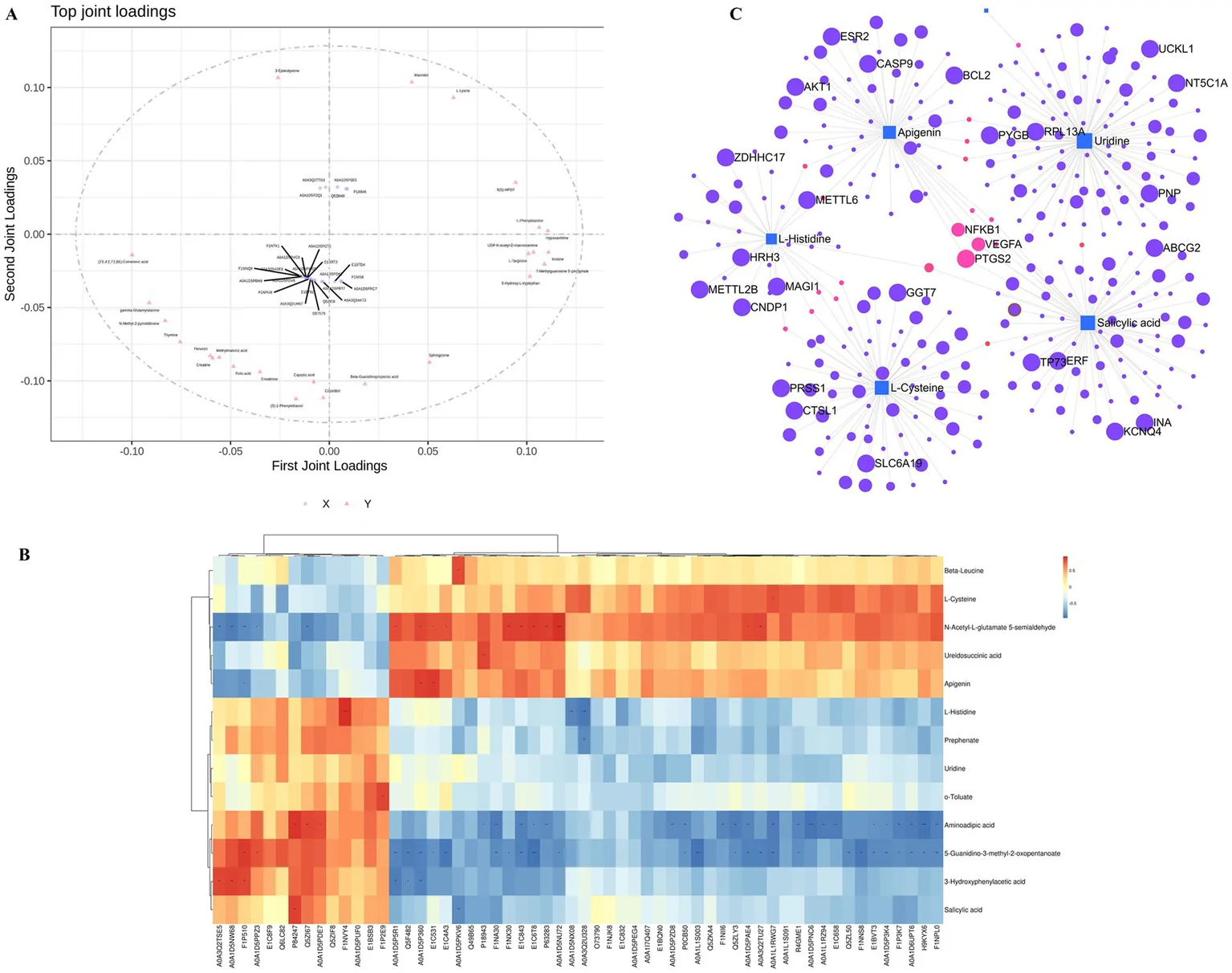
Integrated proteomic and metabolomic analysis of uterine fluid. **(A)** O2PLS score plot showing the correlation between proteomic and untargeted metabolomic datasets derived from the same uterine fluid samples. **(B)** Correlation analysis between differentially abundant proteins (DAPs) and differentially expressed metabolites (DEMs). **(C)** Integrated protein–metabolite interaction network illustrating the relationships between key DAPs and DEMs.

To further investigate molecular associations related to soft-shelled egg formation, an integrated multi-omics analysis was performed using the matched biological samples included in both the proteomic and metabolomic datasets (*n* = 4 per group). The O2PLS model score plot showed an association between the latent components of the proteomic and metabolomic datasets (Figure 6A). Correlation analysis further revealed significant associations between DAPs and DEMs (Figure 6B). Notably, aminoadipic acid and 5-guanidino-3-methyl-2-oxopentanoate exhibited significant negative correlations with the majority of DAPs. Interaction network analysis integrating DAPs and DEMs showed that L-cysteine was associated with SLC6A19 and CTSL1, while L-histidine was associated with CNDP1, suggesting coordinated protein-metabolite relationships within amino acid-related metabolic processes (Figure 6C). Overall, these results indicate that coordinated alterations in proteins and metabolites are associated with amino acid metabolism in the uterine fluid of hens producing soft-shelled eggs.

## 4 Discussion

The pattern observed in the present study suggests that the soft-shelled phenotype was primarily associated with defective shell mineralization rather than a generalized deterioration of internal egg quality. The absence of marked differences in most internal quality traits is consistent with previous reports showing that changes in soft-shelled egg production may occur without parallel changes in egg weight, albumen height, yolk color, or Haugh unit (18). Mechanically, eggshell strength depends not only on total shell thickness but also on the organization of the mammillary and palisade/effective layers and the architecture of the calcite crystals and organic matrix (19, 20). Previous studies have further linked variation in crystal size and orientation, shell ultrastructure, molting, dietary mineral status, shell-gland metabolism, and uterine molecular regulation with eggshell mechanical properties and mineralization (21–26). Benavides-Reyes et al. (27) showed that the age-related loss of eggshell strength was closely associated with changes in mammillary density and calcite crystal organization. Therefore, the reduced mammillary, palisade, and effective layer development observed in the SE group may reflect disturbed crystal nucleation and/or subsequent columnar calcite growth, providing a structural explanation for the extreme fragility of soft-shelled eggs.

Calcium available for eggshell formation is derived from intestinal absorption and skeletal mobilization and is subsequently delivered through the circulation to the shell gland. Villus height, crypt depth, and their ratio are widely used as morphological indicators of intestinal absorptive status in poultry (17, 28–30). In the present study, the lack of group differences in the measured serum calcium-related indicators and duodenal morphology suggests that gross systemic calcium status and intestinal structure were not the major distinguishing features between NE and SE hens at the time of sampling. This does not exclude altered calcium metabolism, because calcium homeostasis also depends on functional regulation of intestinal Ca^2+^ uptake and extrusion, calbindin-mediated transport, and hormone-sensitive calcium-processing pathways (16, 31, 32), which may not be captured by routine serum indices or villus measurements. For example, Wistedt et al. (33) associated deterioration of eggshell quality with reduced shell-gland density and decreased duodenal carbonic anhydrase activity, highlighting the contribution of tissue-level functional regulation to the supply of mineralization precursors. Thus, our findings shift attention from calcium availability per se toward downstream utilization and secretion of mineralization substrates, particularly within the uterus.

The uterus is the final site where Ca^2+^ and HCO₃^−^ are secreted into the uterine fluid and where matrix-guided calcite deposition occurs. The reduced endometrial villus length and mucosal fold height and area observed in SE hens may therefore indicate a reduced effective secretory surface or impaired functional capacity of the uterine mucosa. Reproductive endocrine signaling can also influence laying activity and uterine calcium secretion (34, 35). This interpretation is consistent with Feng et al. (36), who demonstrated that uterine inflammation impaired eggshell thickness and mechanical properties while disrupting calcium transport and matrix protein synthesis, and with Cui et al. (37), who identified ATP2B4-mediated Ca^2+^ extrusion as a regulated component of uterine calcium transport during eggshell calcification. More recently, Fu et al. (38) reported that hens producing low-strength eggs showed uterine tissue damage, impaired calcium transport, slower mammillary and effective layer growth, and deteriorated eggshell ultrastructure. Together, these findings support a mechanism in which compromised uterine structure and ion-secretory capacity may alter local Ca^2+^/HCO₃^−^ delivery and matrix-guided crystal growth, thereby contributing to the thin and poorly organized shell observed in SE hens. However, the present study was cross-sectional and cannot determine whether the uterine morphological alterations precede soft-shelled egg formation or arise as a consequence of the abnormal laying state. Longitudinal or experimental studies are required to establish the direction of this relationship.

In addition to the pronounced structural deterioration of the eggshell, our integrated multi-omics analysis revealed alterations in uterine fluid composition in hens producing soft-shelled eggs. These molecular changes were predominantly enriched in amino acid metabolism and mitochondrial energy-related pathways, suggesting that local metabolic activity within the uterine microenvironment may be involved in eggshell mineralization.

Amino acids serve not only as substrates for protein synthesis but also as regulators of cellular redox balance, energy metabolism, and matrix formation. Among the differentially expressed metabolites, L-cysteine was significantly increased in the SE group and was enriched in pathways such as aminoacyl-tRNA biosynthesis, sulfur relay system, and cysteine and methionine metabolism. L-cysteine is a key sulfur-containing amino acid involved in glutathione synthesis and intracellular redox homeostasis. The increased abundance of L-cysteine in uterine fluid may reflect altered sulfur amino acid metabolism and redox homeostasis in the uterine microenvironment, potentially affecting mitochondrial function and cellular secretory activity (39). Furthermore, aminoacyl-tRNA biosynthesis is essential for translational efficiency and protein production (40). Changes in this pathway suggest that amino acid utilization and protein synthesis may be altered in the uterine fluid of SE hens.

The organic matrix of the eggshell, although constituting only a small proportion of total shell weight, plays an important role in crystal nucleation, orientation, and mechanical strength. Previous proteomic analyses of uterine fluid have identified numerous matrix-related proteins associated with eggshell calcification and shell ultrastructure. For example, Marie et al. (15) demonstrated marked stage-dependent changes in the uterine fluid proteome during eggshell mineralization, with proteins such as ovocleidin-17, ovalbumin, and ovotransferrin showing characteristic abundance patterns during defined stages of calcification. Sun et al. (14) also identified eggshell matrix-specific and calcium-associated proteins in uterine fluid bathing eggs with divergent eggshell mechanical properties. In contrast, classical eggshell matrix proteins were not represented among the 58 DAPs identified in the present study. This discrepancy may partly reflect differences in sampling stage, because uterine fluid in our study was collected after oviposition when no egg was present in the uterus, rather than during active eggshell calcification. Moreover, absence from the DAP list does not necessarily indicate absence from the uterine fluid proteome, as these proteins may not have differed sufficiently in abundance between the NE and SE groups to meet the differential-abundance criteria. Therefore, the protein changes identified here may primarily reflect alterations in the uterine luminal microenvironment associated with the abnormal laying phenotype rather than direct changes in eggshell matrix deposition. Further studies examining uterine fluid at defined stages of active calcification are needed to determine whether classical matrix proteins are differentially regulated during soft-shelled egg formation.

In addition to amino acid-related changes, proteomic data revealed that DAPs were enriched in mitochondrial respiratory chain complexes, ATP synthase components, and mitochondrial membrane-associated proteins. Mitochondria are central to cellular ATP production and ion transport regulation (41). Eggshell mineralization requires transcellular calcium transport and active secretion processes, both of which are energy-dependent. Alterations in mitochondrial protein networks may reflect changes in oxidative phosphorylation-related processes, but mitochondrial function and ATP production were not directly measured in this study.

Integrated protein-metabolite interaction analysis further highlighted coordinated changes within amino acid-related pathways. The associations between L-cysteine and transport-related proteins (e.g., SLC6A19) as well as proteolytic enzymes (e.g., CTSL1) suggest that amino acid transport, turnover, and utilization may be simultaneously altered in the uterine fluid of SE hens. Such coordinated metabolic changes may influence the physicochemical environment required for biomineralization, including pH buffering capacity, ion binding dynamics, and matrix scaffold formation, although these processes require further targeted validation. A limitation of the present study is the relatively small sample size, particularly for the proteomic analysis, which may limit statistical power and the generalizability of the omics findings. Future studies with larger independent cohorts are needed to validate the identified proteins, metabolites, and associated pathways. In addition, soft-shelled and shell-less eggs were not stable, mutually exclusive phenotypes within individual SE hens, which precluded phenotype-specific omics comparisons. Future studies with larger cohorts and phenotype-specific sampling are needed to determine whether these two abnormal egg types involve distinct molecular mechanisms.

This study provides an integrated proteomic and metabolomic view of uterine fluid associated with soft-shelled egg formation and identifies candidate pathways related to amino acid metabolism, mitochondrial energy metabolism, and the uterine microenvironment. Further validation of key proteins and metabolites will help clarify the biological relevance of these pathways and support future studies aimed at improving eggshell quality.

## 5 Conclusion

In summary, soft-shelled eggs showed reduced eggshell thickness, disrupted ultrastructure, and impaired uterine morphology. No significant differences were detected in the measured serum biochemical indicators or duodenal morphology between NE and SE hens. Integrated proteomic and metabolomic analyses revealed changes in uterine fluid composition, mainly involving amino acid metabolism and mitochondrial energy-related pathways. These findings suggest that soft-shelled egg formation is associated with local uterine morphological changes and metabolic changes in the uterine fluid microenvironment, providing candidate pathways for future studies on eggshell calcification and eggshell quality improvement.

## Data Availability

The mass spectrometry proteomics data have been deposited to the ProteomeXchange Consortium via the PRIDE partner repository with the dataset identifier PXD078015. The dataset is available at: https://www.ebi.ac.uk/pride/archive/projects/PXD078015. The data that support the findings of this study are available from the corresponding author upon reasonable request.
